# Influence of surgical stabilization of clavicle fractures in multiply-injured patients with thoracic trauma

**DOI:** 10.1038/s41598-021-02771-5

**Published:** 2021-12-01

**Authors:** Helge Eberbach, Rolf Lefering, Sven Hager, Klaus Schumm, Lisa Bode, Martin Jaeger, Dirk Maier, Johannes Kalbhenn, Thorsten Hammer, Hagen Schmal, Jörg Bayer

**Affiliations:** 1 Department of Orthopaedic and Trauma Surgery, Medical Center, Faculty of Medicine, University of Freiburg, Hugstetter Straße 55, 79106 Freiburg, Germany; 2 IFOM-Institute for Research in Operative Medicine, University Witten/Herdecke, Faculty of Health, Cologne, Germany; 3 Department of Surgery, Bautzen Hospital, Oberlausitz-Kliniken gGmbH, Bautzen, Germany; 4 Department of Anesthesiology and Intensive Care Medicine, Medical Center, Faculty of Medicine, University of Freiburg, Freiburg, Germany; 5 Department of Orthopaedic Surgery, University Hospital Odense, Odense C, Denmark

**Keywords:** Diseases, Trauma

## Abstract

Thoracic trauma has decisive influence on the outcome of multiply-injured patients and is often associated with clavicle fractures. The affected patients are prone to lung dysfunction and multiple organ failure. A multi-center, retrospective analysis of patient records documented in the TraumaRegister DGU was performed to assess the influence of surgical stabilization of clavicle fractures in patients with thoracic trauma. A total of 3,209 patients were included in the analysis. In 1362 patients (42%) the clavicle fracture was treated operatively after 7.1 ± 5.3 days. Surgically treated patients had a significant reduction in lung failure (p = 0.013, OR = 0.74), multiple organ failure (p = 0.001, OR = 0.64), intubation time (p = 0.004; −1.81 days) and length of hospital stay (p = 0.014; −1.51 days) compared to non-operative treatment. Moreover, surgical fixation of the clavicle within five days following hospital admission significantly reduced the rates of lung failure (p = 0.01, OR = 0.62), multiple organ failure (p = 0.01, OR = 0.59) and length of hospital stay (p = 0.01; −2.1 days). Based on our results, multiply-injured patients with thoracic trauma and concomitant clavicle fracture may benefit significantly from surgical stabilization of a clavicle fracture, especially when surgery is performed within the first five days after hospital admission.

## Introduction

More than 50% of all multiply-injured patients with an injury severity score ≥ 16 suffer from thoracic trauma^1,2^. Early detection of life-threatening injuries and administration of optimal emergency care (e.g. chest tube, intubation, fluid administration) are essential to achieve the best possible outcome^3,4^. However, late or inadequate treatment of thoracic trauma increases the risk of developing severe complications such as respiratory failure, ARDS, long-term morbidity, and higher mortality rates^3,5–7^.

A reliable indicator for underlying thoracic injuries is a clavicle fracture which is easily diagnosed during the primary survey in early trauma patient care^8^. In multiply-injured patients sustaining a clavicle fracture concomitant thoracic injuries occur in 77%^9^. Therefore, the clavicle can be considered the gatekeeper of the thorax^10,11^.

Despite advances in ventilatory management like lung-protective, non-invasive ventilation protocols, patients with thoracic trauma often require prolonged ventilatory support and protracted ICU and hospital stays^12–14^. Since clavicular injuries represent a severe loss of integrity of the chest and upper quarter of the body, disorders in breathing mechanics can occur^15^. This might be attributed to either direct functional deficiency or as a consequence of associated pain^15^, especially since the breathing mechanism does not only consist of the chest wall itself and the diaphragm. An intact and stable clavicle, as the origin of the accessory inspiratory muscles, is a crucial prerequisite for effective respiration and oxygenation^16,17^.

Therefore, the purpose of this study was to investigate the influence of surgical stabilization of clavicular fractures in multiply-injured patients with thoracic trauma on lung failure, multiple organ failure, sepsis rate, intubation time, length of intensive care unit stay and length of hospital stay. We hypothesized that osteosynthesis of the clavicle is associated with reduced complication rates and has an influence on ICU and hospital stay.

## Results

### Basic characteristics of all included patients

**Figure 1:**
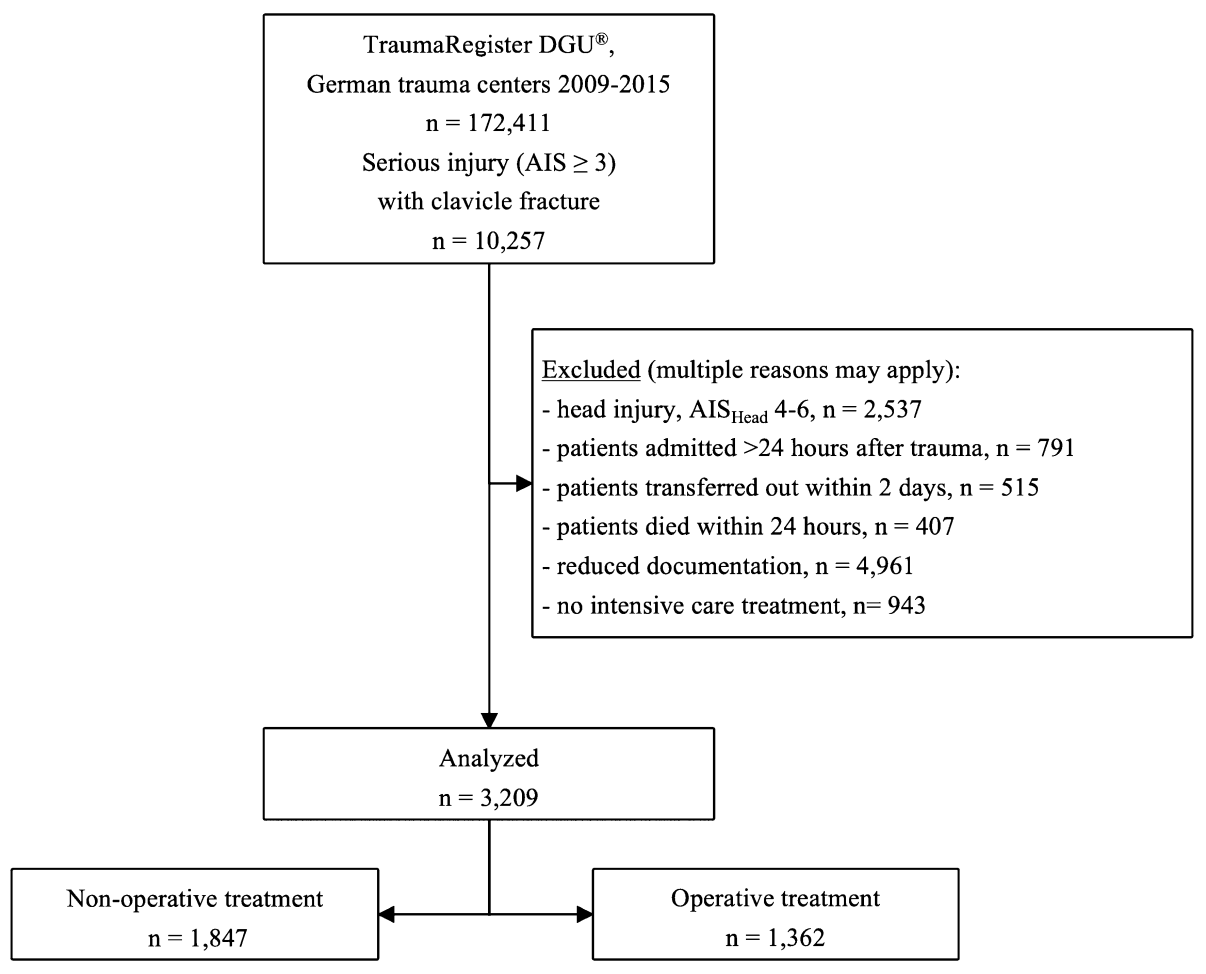
Flowchart showing inclusion and exclusion of patients for this study.

The initial database contained 145,518 patients. After applying inclusion and exclusion criteria a total of 3,209 multiply-injured patients with thoracic trauma and concomitant clavicle fracture remained for analysis (Fig. 1).

The included patients were predominantly male (73.5%), had a mean age of 49.0 ± 19.3 years and a mean ISS of 22.9 ± 8.8. In 1362 patients (42.0%) the clavicular fracture was treated operatively after 7.1 ± 5.3 days. 19.1% had an AIS_Thorax_ < 3 and 80.9% an AIS_Thorax_ ≥ 3. The overall mortality rate was 3.7%; 6.1% of patients deceased in the non-operative group compared to 0.6% in the operative group. Further study group’s characteristics are summarized in Table 1.

**Table 1:** Basic characteristics.

	n	Sex (f/m)	Age (years)	ISS	Number of injuries (n)	Mortality
Non-operative treatment	1847 (57.6%)	28.4/71.6%	51.3 ± 20.8	22.6 ± 9.1	6.8 ± 3.0	6.1%
Operative treatment	1362 (42.4%)	24.0/76.0%	45.9 ± 16.6	21.3 ± 8.3	6.4 ± 2.8	0.6%
Total	3209 (100%)	26.5/73.5%	49.0 ± 19.3	22.0 ± 8.8	6.6 ± 2.9	3.7%
Significance		0.005*	< 0.001*	< 0.001*	0.001*	< 0.001*

*ISS* injury severity score; number of injuries, conditions that coexisted at the time of admission and caused by the trauma.

*p < 0.05.

**Figure 2:**
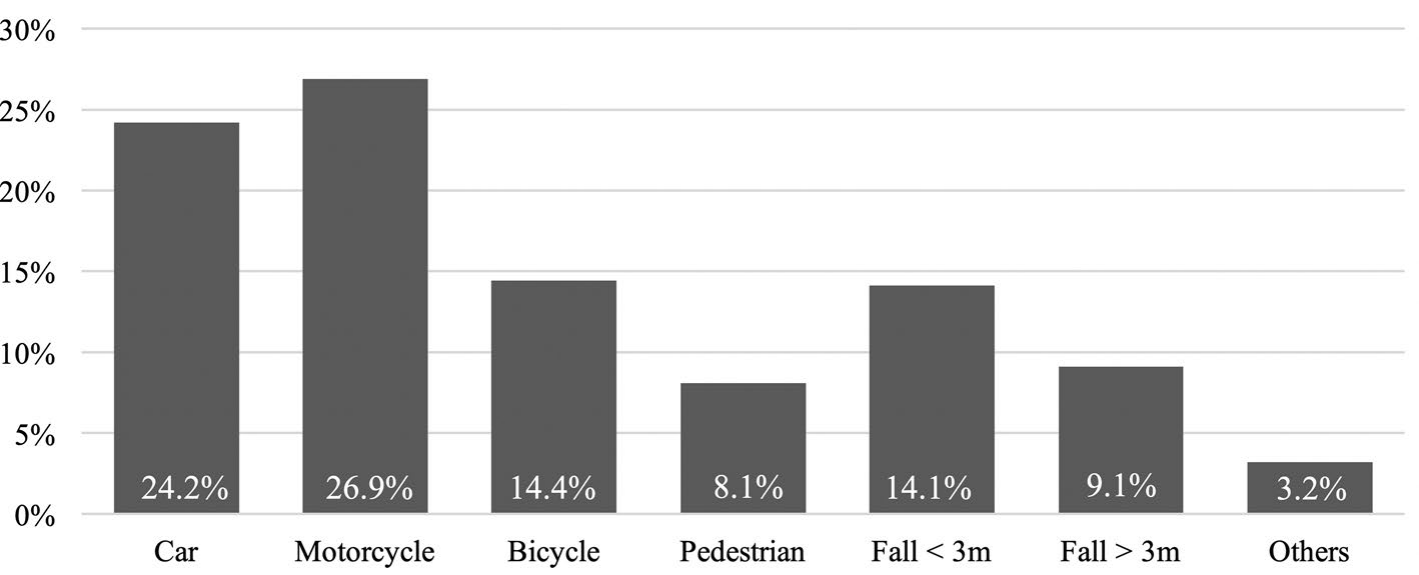
Trauma mechanism.

The majority of patients sustained their injuries during road traffic accidents, mainly riding motorcycles (26.9%) and cars (24.2%). Figure 2 illustrates all accident causes.

Most patients (83.2%) were treated at a supra-regional level I trauma center, 15.1% at a regional level II center and 1.7% at a local level III trauma center.

**Figure 3:**
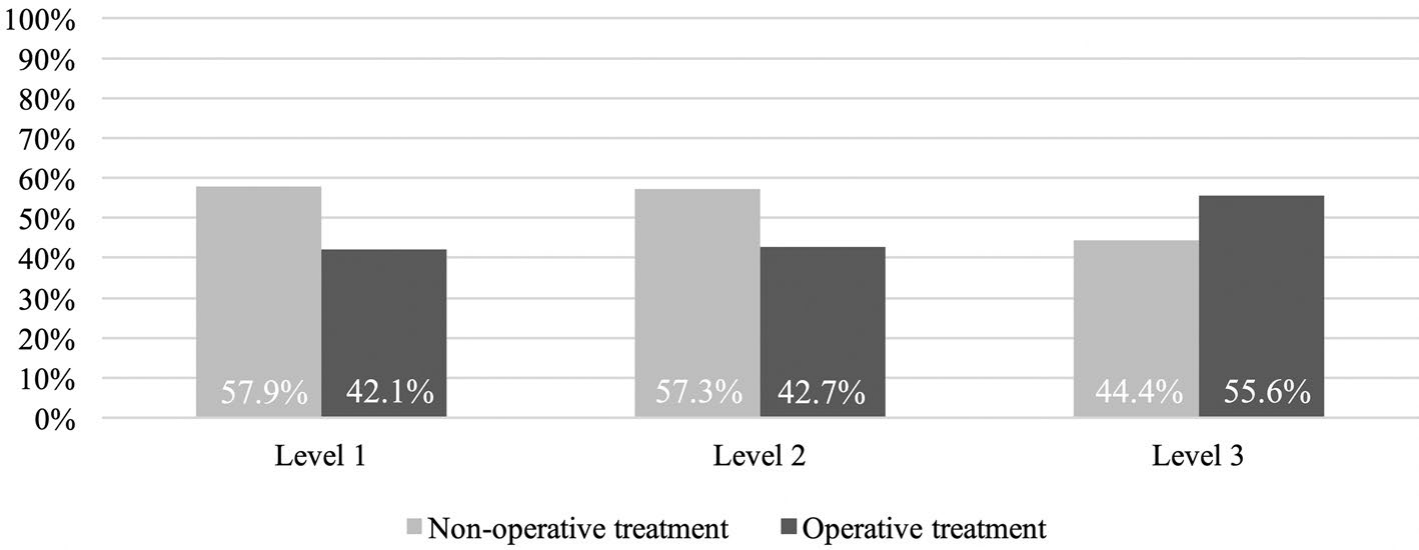
Treatment modality according to level of trauma center.

In level I and II centers patients received non-operative treatment in the majority of cases (57.9% and 57.3%, respectively), whereas in level III trauma centers operative therapy was chosen more frequently (55.6%), as shown in Fig. 3.

**Figure 4:**
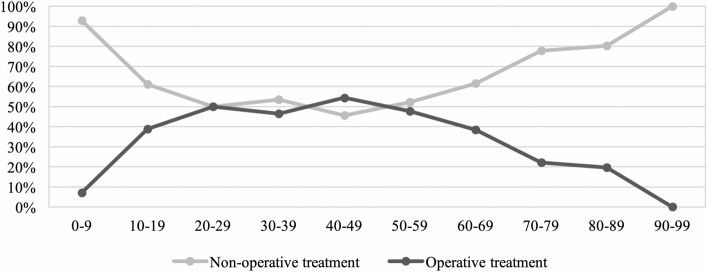
Treatment modality according to patient age.

Overall, non-operative therapy was preferred especially in patients under 20 and over 60 years of age (Fig. 4).

19.3% of finally analyzed patients suffered from subsequent lung failure, 19.7% from multiple organ failure and 6.4% sustained sepsis. For all included patients, mean time spent on ICU was 9.4 ± 11.7 days, mean intubation time 7.4 ± 11.4 days and mean hospital stay 22.4 ± 18.0 days.

Patients with operative treated clavicle fractures also seemed to have lower rates of lung, multiple organ failure and sepsis and spent less time intubated in ICU as well as in hospital (Table 2).

**Table 2:** Outcome parameters.

	LF	MOF	Sepsis	ICU (days)	Intubation (days)	Hospitalization (days)
Non-operative treatment	23.1%	24.8%	7.3%	10.5 ± 12.4	8.7 ± 12.0	22.6 ± 18.1
Operative treatment	14.1%	12.9%	5.1%	7.9 ± 10.4	5.3 ± 10.4	22.0 ± 17.9
Total	19.3%	19.7%	6.4%	9.4 ± 11.7	7.4 ± 11.4	22.4 ± 18.0

*LF* lung failure, *MOF* multiple organ failure, *ICU* intensive care unit.

Whether these differences were statistically significant and attributable to operative treatment we sought to examine with the following logistic regression analysis.

### Influence of operative treatment on outcome parameters

In the regression analysis surgically treated patients showed significantly reduced rates of lung failure (p = 0.013, OR = 0.74, [0.58–0.94]) and multiple organ failure (p = 0.001, OR = 0.64, [0.50–0,83]).

While no significant effect was found on sepsis rates and time on ICU, operatively treated patients had significant less intubation time (p = 0.004; −1.8 days) and less total days in hospital (p = 0.014; −1.5 days) compared to non-operatively treated patients (Table 3).

**Table 3:** Influence of operative treatment on outcome parameters.

	LF	MOF	Sepsis	ICU (days)	Intubation (days)	Hospitalization (days)
Regression coefficient	−0.23	−0.44	−0.08	−0.53	−1.81	−1.51
Odds ratio [95% CI]	0.74 [0.58–0.94]	0.64 [0.50–0.83]	0.92 [0.63–1.33]	–	–	–
Significance	0.013*	0.001*	0.669	0.182	0.004*	0.014*

Regression model includes: age, sex, ISS, AIS_Head_, AIS_Thorax,_ AIS_Abdomen_, AIS_Extremities_, shock (< 90 mmHg) in the trauma room, blood transfusion, intubation.

*LF* lung failure, *MOF* multiple organ failure, *ICU* intensive care unit.

*p < 0.05.

### Influence of early operative treatment (≤ 5 days) on outcome parameters

To assess a possible effect of early operative intervention within the first 5 days after hospital admission within the subgroup of 620 surgically treated patients, we performed an additional logistic regression analysis.

When considering the operatively treated patients only, early surgical fixation of the clavicle within the first five days led to significant reduction of lung failure (p = 0.01, OR = 0.62, [0.44–0.88]) multiple organ failure (p = 0.01, OR = 0.59, [0.41–0.86]) and total days spent in hospital (p = 0.01; −2.1 days) (Table 4) compared to operative treatment after 5 days.

**Table 4:** Influence of early operative treatment (≤ 5 days) on outcome parameters.

	LF	MOF	Intubation (days)	Hospitalization (days)
Regression coefficient	−0.47	−0.53	−1.84	−2.10
Odds ratio [95% CI]	0.62 [0.44–0,88]	0.59 [0.41–0,86]	–	–
Significance	0.008*	0.006*	0.534	0.007*

Regression model includes: age, sex, ISS, AIS_Head_, AIS_Thorax,_ AIS_Abdomen_, AIS_Extremities_, shock (< 90 mmHg) in the trauma room, blood transfusion, intubation.

*LF* lung failure, *MOF* multiple organ failure.

*p < 0.05.

## Discussion

The most important finding of our present retrospective study evaluating 3209 multiply-injured patients is that operative stabilization of concomitant clavicle fractures in patients with thoracic trauma has decisive positive effects on clinically relevant outcome parameters.

In addition to the serious, life-threatening thoracic organ injuries, the bony chest wall is at great risk in multiply-injured patients^18^. Affection of the clavicle is the third most common fracture in multiply-injured patients (10.4%), only surpassed by femoral (16.5%) and tibial (12.6%) fractures^19^. Regarding all multiply-injured patients with bony injuries to the chest wall, an additional clavicular fracture is present in even 18.8% of the cases^20^. Thus, clavicle fractures are common injuries in patients suffering from thoracic trauma.

Basic characteristics of our study cohort correspond to other studies describing the epidemiology of clavicle fractures in thoracic trauma, like the Dutch National Trauma Database^11^ or a British collective from Leeds^21^. In these populations patients were also predominantly male and had a mean age of 39.1 and 47.5 years, respectively. While our reported mean age of 49.0 years is slightly higher, their reported ISS was higher with 28.7 and 29.2, compared to our mean ISS of 22.9^11,21^.

Similar to previously published data on thoracic trauma, motorcycle crashes could be identified as the most frequent injury mechanism for developing a severe thoracic trauma with a clavicle fracture^11,14,22,23^.

One concern in most studies dealing with multiply-injured patients suffering from thoracic trauma is the heterogeneous patient collective regarding the leading injury and the immanent implication on the intensive care course (e.g. periods of mechanical ventilation in severe head trauma). By deliberately excluding more than moderate brain injuries in our patient collective, we sought to limit this risk of bias.

It has been suggested that patients sustaining serious trauma to the chest and suffering from three or more rib fractures should be transferred to a high-volume level 1 trauma center^24,25^. In line with this recommendation, our patients were treated mainly at level 1 and level 2 trauma centers (98.3%).

In 42.0% of cases the clavicular fracture was treated operatively. A study with older data reviewing the years 2002–2013 in the TraumaRegister DGU^®^ described an even higher rate of 52.4% operative treatments^26^, which might be due to more generous inclusion criteria, as all patients with an ISS ≥ 16 were included.

Our reported patients suffering from thoracic trauma and clavicle fracture showed a prolonged period of ICU stay (mean 9.4 days) and intubation time (mean 7.4 days) compared to published multiply-injured patients without thoracic trauma (ICU: 8.1 days, intubation time: 3.9 days)^3^. Yet, our findings are comparable with previously published data on patients with thoracic trauma (ICU: 11 days, intubation time: 7 days^27^; ICU: 12 days, intubation time: 7 days^14^).

In expert opinions and small case series, several authors recommend surgical stabilization of clavicular fractures in thoracic trauma, particularly in the case of displaced fractures and associated serial rib fractures or flail chest injuries^10,28^. However, to the best of our knowledge, the implication of surgical management of clavicle fractures in thoracic trauma on clinical outcome parameters had yet to be evaluated. This is therefore the first study addressing the impact of operative treatment of the clavicle fracture in patients with relevant thoracic trauma on outcome parameters in a large collective. There are various theoretical reasons supporting surgical stabilization in this subgroup.

In patients with high-energy mechanism of injury clavicle fractures are prone to fracture displacement, also when initial imaging shows nondisplacement^29,30^.

Since the clavicle acts as a stabilizer of the upper quadrant of the chest fracture displacement is causing pain, especially in patients with concomitant ipsilateral rib fractures. The skeleton of the shoulder and chest loses stability and can lead to a loss of function of the shoulder and a pronounced deformation of the chest wall^10^.

In particular, the breathing mechanism is affected. A fractured and instable clavicle as the origin of the accessory inspiratory muscles (M. sternocleidomastoideus, Mm. scaleni, M. pectoralis) can cause an ineffective respiration and oxygenation^16,17^.

In combination with a severe thoracic trauma it can thereby further the development of multiple organ dysfunction and pulmonary failure^31,32^. Furthermore, it is well known that 80% of patients with multiple organ failure start with lung failure^21^, and that severe thoracic trauma is an independent risk factor for developing multiple organ failure^33,34^.

To support the thesis that surgical fixation of fractures to the thoracic wall (or origins of inspiratory muscles) can support patient recovery in thoracic trauma several studies of rib fracture fixation have been published. Concerning flail chest injuries there is valid data supporting operative care of the injury to stabilize the thoracic aperture. Flail chest occurs when three or more adjacent ribs are fractured in at least two places, creating a chest wall segment that moves paradoxically from the chest wall^35^, The operative treatment of flail chest injuries shows a clear advantage with regard to the clinical course and outcomes in polytrauma^13,35^. According to the metaanalysis of Leinicke et al. patients with early stabilization have less intubation time and both the length of stay in the intensive care unit and the total length of stay in the hospital could be significantly reduced by surgical therapy^13^.

In our present study we are the first to demonstrate similar results concerning surgical stabilization of clavicle fractures.

80.9% of our patients suffered from at least serious thoracic trauma (AIS_Thorax_ ≥ 3). Therefore, to analyze the effect of operative treatment of the clavicle fracture for all thoracic trauma patients the independent variable AIS_Thorax_ was included into to the regression model. We also included other possible confounders (age, sex, ISS, AIS_Head_, AIS_Thorax_, AIS_Abdomen_, AIS_Extremities_, shock RR_syst_ < 90 mmHg, intubation and blood transfusion) as independent variables into the regression model, and therefore sought to control for confounding.

Surgically treated patients showed a significantly reduced intubation time and length of hospital stay compared to conservative care. Furthermore, the risk of lung failure and multiple organ failure was significantly reduced. Mortality was eminently lower in the operative subgroup. Nevertheless, the reduction in mortality in our data should not be overestimated, because combinations of injuries or comorbidities with a clinically relevant likelihood of mortality can be a reason for not undergoing surgery and therefore pose a bias towards higher mortality in the conservatively treated subgroup.

In our study, the influence of surgical clavicle stabilization on sepsis and length of ICU-stay was not statistically significant. One reason for that finding could be that these variables might be eminently influenced by other major injuries besides the thoracic trauma.

According to our results, operative management of clavicle fractures in thoracic trauma might therefore be a promising treatment strategy that improves patient’s outcome, and also lower treatment costs due to less complications and total days in hospital. Yet, these positive results come with the cost of an operative intervention.

But, complications of surgical treatment of clavicle fractures are rare. In a retrospective review of 1350 clavicular internal fixations, by plate or intramedullary fixation, Leroux et al. reported neurovascular complications to be exceptional, with only 5 neurologic and 5 vascular complications: i.e., < 1%^36^. A systematic review of Rehn et al. found a prevalence of complications that required additional major surgery of 3.3% in the operative groups compared to 8.6% in the non-operative groups^37^.

As shown in other studies surgical timing and early operative treatment may result in positive implications for the intensive care unit stay and clinical outcome^38,39^. In line with studies showing benefits in organ failure reduction and shortening of intubation periods after early operative treatment of fractures in different body regions we are the first to describe this association in early stabilization of clavicle fractures in thoracic trauma patients.

This study is limited by the nature of a retrospective registry study. All findings represent associations and do not claim any causality. Registry data are less valid than data obtained in a prospective randomized trial setting. The comparability of groups is questioned since baseline characteristics are different. Furthermore, it might be the case that the operative treatment itself could be an indirect marker for a relative stable situation of the patient. However, the clavicle ORIF is a minor intervention, and the decision to operate or not is not as affected as it is for major surgeries. The clinical relevance, as well as the influence on the outcome, has to be evaluated in future clinical studies.

Hospitals participating in the TR-DGU^®^ are regularly audited, and sample tests are taken to ensure data quality. However, the validity of their documentation is not verified by external monitors as in prospective trials^40^.

To minimize confounding, we excluded patients with more than moderate head injury (AIS ≥ 3) in our study and, as a result, our findings cannot be readily transferred to severely injured patients sustaining additional major trauma to this body region. We are not able to comment on the performed surgical procedures (e.g. plate osteosynthesis, elastic stable intramedullary nailing, etc.) and treatment protocols, since this information is not available in the TR-DGU^®^ database.

Additionally, we are unable to comment on the fracture morphology, displacement and fractured part of the clavicle, since this information is not available in the TR-DGU® database, either. Therefore, we were unable to include this information in our statistical regression model.

There is an ongoing discussion on the management of multiply-injured patients and timing of osteosynthesis^38^. For small bone fractures no recommendations or studies exist on when to surgically stabilize these injuries in multiply injured patients. For long bone and spinal fractures research exists and some favor stabilization within 72 h after hospital admission^38,39^. Since long bone and spinal fractures are more relevant to patient management on the ICU and are preferably stabilized before small bone fractures, we concluded to examine the time for early clavicular stabilization until 5 days after hospital admission. Although, as mentioned above, this timing is chosen in lack of scientific evidence we based our decision on reasonable clinical experience.

Besides the above stated limitations, to the best of our knowledge, we are the first study group to present comprehensive data on clavicle surgery and timing in multiply- injured patients suffering from concomitant thoracic trauma.

Based on our data, surgical stabilization of clavicle fractures in multiply-injured patients with thoracic trauma may be beneficial and significantly reduces lung and multiple organ failure rates as well as time of invasive ventilation and length of hospital stay. The effect of reduced lung und multiple organ failure rates, as well as shorter hospital stay, was significantly associated with the clavicle stabilization being performed within 5 days of hospital admission. Further studies regarding patient-tailored surgery are warranted to elucidate which multiply-injured patients profit from clavicular stabilization.

## Methods

### The TraumaRegister DGU®

The TraumaRegister DGU® (TR-DGU) of the German Trauma Society (DGU) was founded in 1993. The aim of this multicenter database is the anonymized and standardized documentation of severely injured patients.

Data are collected prospectively in four consecutive time periods from the accident site until hospital discharge: (A) pre-hospital phase, (B) emergency room and initial surgery, (C) ICU, and (D) discharge. The documentation includes detailed information on demographics, injury patterns, comorbidities, pre- and in-hospital management, course in the ICU, relevant laboratory findings including each individual’s data on transfusions, and outcome.

The inclusion criterion is admission to the hospital via the emergency room with subsequent ICU care or reaching the hospital with vital signs and dying before admission to the ICU.

The infrastructure for documentation, data management, and data analysis is provided by the Academy for Trauma Surgery (AUC), a company affiliated with the German Trauma Society.

Scientific leadership is provided by the Committee on Emergency Medicine, Intensive Care and Trauma Management (Section NIS) of the German Trauma Society. The participating hospitals submit their anonymized data into a central database via a web-based application. The quality of the scientific data analysis is monitored by peer review procedure established by Section NIS.

The participating hospitals are primarily located in Germany (90%), but a rising number of hospitals in other countries are contributing data as well (currently from Austria, Belgium, China, Finland, Luxemburg, Slovenia, Switzerland, The Netherlands, and the United Arab Emirates). Approximately 35,000 cases from more than 700 hospitals have been entered into the database per year.

Participation in TR-DGU is voluntary. For hospitals associated with TraumaNetzwerk DGU®, however, the entry of at least a basic data set is obligatory for reasons of quality assurance. Hospitals interested in trauma research must enter a standard data collection form that contains more comprehensive information (e.g., organ failure, sepsis) on the patient course compared with the basic data set.

### Patients

Patients documented between 2009 and 2015 in the TR-DGU® were analyzed for eligibility in this investigation. Patient selection based on the following criteria: (1) standard documentation (including surgery and organ failure assessment) from German trauma centers, (2) diagnosed clavicle fracture with documentation of treatment modality (3) maximum AIS severity ≥ 3 and (4) requirement of initial intensive care.

Patients with relevant head injury, defined as AIS_Head_ 4–6, were excluded since serious head trauma may pose an indication for intubation and prolonged mechanical ventilation itself^41^. Also, patients admitted more than 24 h after trauma to a referring hospital and patients transferred into another institution within 2 days after hospital admission were excluded to prevent confounding. Finally, patients who died within 24 h were excluded from analysis since the surgical treatment of their clavicle fracture was deemed unlikely (Fig. 1).

Patients included in the final analysis were divided into two subgroups (operative versus non-operative treatment) according to the clavicle fracture treatment. The operative therapy group comprises all stabilization methods performed (e.g. plate osteosynthesis, elastic stable intramedullary nailing, etc.).

We performed an additional subgroup analysis of patients undergoing surgical clavicular stabilization regarding the timing of surgery to evaluate the impact of early operation. Therefore, we defined the time frame for early clavicular surgery to be performed within 5 days after trauma.

Injuries were graded according to the 2008 version of the Abbreviated Injury Scale (AIS)^42^, and the injury severity score (ISS)^43^ was calculated as described. The ISS is calculated from the three worst affected body regions as the sum of squares of the respective AIS severity levels^44^.

Lung failure was assessed by the sepsis-related organ failure assessment (SOFA) score. The SOFA score describes organ function in the respiratory, cardiovascular, renal, hematologic, hepatic, and central nervous systems^45^. Each organ system was graded to evaluate the severity of organ dysfunction or failure. Patients with organ failure entered in the TR-DGU database had to have met the SOFA score criteria for organ failure (3 or 4 points per organ) for at least 2 days.

Multiple organ failure (MOF) and sepsis were assessed according to published guidelines^46^. Sepsis was defined as a systemic response to infection (proven presence of microorganisms). Data on respiratory failure, MOF, and sepsis were only available from patients documented with the standard data collection form.

Length of intensive care unit stay was defined as days spent in the intensive care unit and length of mechanical ventilation was defined as the number of days spent in the ICU with endotracheal intubation or tracheostomy and mandatory mechanical ventilation (e.g. excluding non-invasive ventilation). Length of hospital stay was defined as the time spent in the hospital.

The Revised Injury Severity Classification II Score (RISC II)^47^ was calculated for outcome adjustment. The model consists of the following predictors: worst and second-worst injury (AIS severity level), head injury, age, sex, mechanism, pupil reactivity and size, pre-injury health status, blood pressure, acidosis (base deficit), coagulation, hemoglobin, and cardiopulmonary resuscitation^47^.

### Statistical analysis

Demographic and clinical characteristics comparing two treatment groups were evaluated using descriptive statistics. Continuous variables are presented as mean with standard deviation (SD), while categorical variables are presented as number of cases with percentages. The respective statistics refer to patients with valid data sets only. Data of lung failure, MOF and sepsis are not part of the basic data set. Therefore, the total number of patients or characteristics may vary.

Statistical testing for the effect of operative treatment on the outcome was performed using multivariate logistic or linear regression analysis. In this analysis, the dependent variables were lung failure, MOF, sepsis, length of ICU stay, length of mechanical ventilation and length of hospital stay, respectively. The independent variables consisted of patient characteristics (age, sex), injury severity (ISS, AIS_Head_, AIS_Thorax_, AIS_Abdomen_, AIS_Extremities_), hemodynamic parameters (shock defined as RR_syst_ ≤ 90 mmHg on admission) and therapeutic interventions (intubation, blood transfusion).

A subgroup-analysis was performed to assess the impact of early operative treatment within five days after trauma compared to late operative treatment.

The level of significance was set at p < 0.05. All data were analyzed using SPSS, version 26.0 (IBM Corp. in New York, USA).

### Ethics declarations

The present study is in line with the publication guidelines of the TR-DGU® and registered as TR-DGU® project ID 2017-006. The study was approved by the University of Freiburg Ethics Committee (EK 167/20) and informed consent was waived. All research was performed in accordance with relevant guidelines/regulations.

## Data Availability

The datasets used and/or analyzed during the current study are available from the corresponding author on reasonable request.

## Abbreviations

AIS: Abbreviated injury scale
ARDS: Acute respiratory distress syndrome
ICU: Intensive care unit
ISS: Injury severity score
MOF: Multiple organ failure
LF: Lung failure
OR: Odds ratio
RISC: Revised Injury Severity Classification
RR_syst_: Systolic blood pressure
SD: Standard deviation
SOFA: Sepsis-related organ failure assessment
TR-DGU^®^: TraumaRegister DGU^®^
